# Supplementary material for: Structure of the Mating-Type Genes and Mating Systems of Verpa bohemica and Verpa conica (Ascomycota, Pezizomycotina)
Source: J Fungi (Basel). 2023 Dec 15;9(12):1202. doi: 10.3390/jof9121202 (PMC10745113; doi:10.3390/jof9121202)
Supplement: Supplementary file 1 [file jof-09-01202-s001.zip › supplementary materials/Supplementary Figure S1.pdf]

Consensus

1. *MATI-1-1 V. bohemica* strain No. 20020 Gene05694
2. *MATI-1-1 V. bohemica* strain No. 20124 Gene05692
3. *MATI-1-1 V. bohemica* strain No. 21108 Gene05691
4. *MATI-1-1 V. conica* strain No. 21110 Gene05311

```
   MAFRPVFLXELVRANPPXXXXXSXXXDPMKRALALRNATLGCALYLAESHDYVLMQDNTGGVWVQHKRSSIIVXPPEGLI
1  MAFRPVFLSELVRANPPFPAYFSIYNDPMKRALALRNATLGCALYLAESHDYVLMQDNTGGVWVQHKRSSIIVAPPEGLI  80
2  ---------------------------MKRALALRNATLGCALYLAESHDYVLMQDNTGGVWVQHKRSSIIVAPPEGLI  52
3  MAFRPVFLSELVRANPPFPAYFSICNDPMKRALALRNATLGCALYLAESHDYVLMQDNTGGVWVQHKRSSIIVTPPEGLI  80
4  MAFRPVFLTELVRANPPSQTHISMCIDPMKRALALRNATLGCALYLAEVHDYILMQDRTGGVWVQHKKSPMIMTPPGGLI  80

   FRVKGESLPYQRWLSSMHKVVRQQGRTGLDPNLSFRCGVERLYSDHFKDKKFTKXFSIGVLSKRSALKALNPYIAHRTWI
1  FRVKGESLPYQRWLSSMHKVVRQQGRTGLDPNLSFRCGVERLYSDHFKDKKFTKHFSIGVLSKRSALKALNPYIAHRTWI  160
2  FRVKGESLPYQRWLSSMHKVVRQQGRTGLDPNLSFRCGVERLYSDHFKDKKFTKHFSIGVLSKRSALKALNPYIAHRTWI  132
3  FRVKGESLPYQRWLSSMHKVVRQQGRTGLDPNLSFRCGVERLYSDHFKDKKFTKDFPIGVLSKRSALKALNPYIAHRAWI  160
4  FKVKGEPLPYQRWLCSMHKAVRQQGRTGLDPKLSFRRGVQRLYGDHFKDNQPTKTQSLGPTSKRSALKALNPYIAHRTWI  160

   SKFLGGLGFTQMSISSLTKGLWKRETRKALWSNIARLYTYARNEDQQGLVLGDFIMSELXRNNQDXSPRTFLLACGIDID
1  SKFLGGLGFTQMSISSLTKGLWKRETRKALWSNIARLYTYARNEDQQGLVLGDFIMSELMRNNQDRSPRTFLLACGIDID  240
2  SKFLGGLGFTQMSISSLTKGLWKRETRKALWSNIARLYTYARNEDQQGLVLGDFIMSELMRNNQDRSPRTFLLACGIDID  212
3  SKFLGGLGFTQMSISSLTKGLWKRETRKALWSNIARLYTYARNEDQHGLVLGDFIMSELTRDNQDPSPRTFLLACGIDID  240
4  SKFLGGLGFTQMVISSLTKGLWQRETRKALWSNIARLYTYARNEDQQGLVLGDFMRAELIRNNQDPSPRTFLLGCGINID  240

   XMREKLDPDYCKFDAQITTPNISXXXXX---XXXXX------XXXXAP----IEPVYPRTTMVSXIYFKPPQNIXTSEQN
1  GMREKLDPDYCKFDAQITTPNIS-----------------------AP----IEPVYPRTTMVSEIYFKPPQNIKTSEQN  293
2  GMREKLDPDYCKFDAQITTPNIS-----------------------AP----IEPVYPRTTMVSEIYFKPPQNIKTSEQN  265
3  RMREKLDPDYCKLDAQITTPNISTPDYCKFDAQITI------PNIPAP----IEPVYPRTTMVSGIYFKPPQNIETSEQN  310
4  EMRKQLVPNCSKFDAQMTIPSIPTPSIA---APITIAPITIAPNISAPNTLALEWAYPRTTVVSNVYFTKPANIEIHEEL  317

   DFGATPIPYTXVSPMNILTNAXSNSI-FYFEEFXPXX---
1  DFGATPIPYTNVSPMNILTNATSNSI-FYFEEFTPEP---  329
2  DFGATPIPYTNVSPMNILTNATSNSI-FYFEEFTPEP---  301
3  GFGATPIPYTDVSPMNILTNAASDSI-FYFEGDSC-----  344
4  D--TLQLSYVDFNPMDSMPISVANEYSFHFEEFVPKSPYA  355
```
