# Supplementary material for: Structure of the Mating-Type Genes and Mating Systems of Verpa bohemica and Verpa conica (Ascomycota, Pezizomycotina)
Source: J Fungi (Basel). 2023 Dec 15;9(12):1202. doi: 10.3390/jof9121202 (PMC10745113; doi:10.3390/jof9121202)
Supplement: Supplementary file 1 [file jof-09-01202-s001.zip › supplementary materials/Supplementary Figure S2.pdf]

Consensus

1. *MATI-2-1 V. bohemica* strain No. 20020 Gene05693
2. *MATI-2-1 V. bohemica* strain No. 20124 Gene05691
3. *MATI-2-1 V. bohemica* strain No. 21108 Gene05690
4. *MATI-2-1 V. conica* strain No. 21117 Gene05375
5. *MATI-2-1 V. conica* strain No. 21120 Gene05382

```
   MPSSXXPXQMLREAIGPINQRFGYINEQNALNKLAGFRIEYDPSFDFVGSDGYTFSVKVFTTSDPNKSLELGDVNKPETY
1  ---------MLREAIGPINQRFGYINEQNALNKLAGFRIEYDPSFDFVGSDGYTFSVKVFTTSDPNKSLELGDVNKPETY  71
2  MPSSLDPFQMLREAIGPINQRFGYINEQNALNKLAGFRIEYDPSFDFVGSDGYTFSVKVFTTSDPNKSLELGDVNKPETY  80
3  MPSSLDPFQMLREAIGPINQRFGYINEQNALNKLAGFRIEYDPSFDFVGSDGYTFSVKVFTTSDPNKSLELGDVNKPETY  80
4  ---------MLREAIWHINQRFSYINEQNALNKLAGFRIEYDPSFDFIGSDGNTFSVKVFTTPDPNKSLELGDISEPETY  71
5  MPSSIQPAQMLREAIWHINQRFSYINEQNALNKLAGFRIEYDPSFDFIGSDGNTFSVKVFTTPDPNKSLELGDISEPETY  80

   TVLTKTGFIRNYPCIRTNEGALIFVHDPATTGADKIYEGYKKFIDPQ---------------------XXXFIIYRNAEM
1  TVLTKTGFIRNYPCIRTNEGALIFVHDPATTGADKIYEGYKKFIDPQ---------------------TSSFIIYRNAEM  130
2  TVLTKTGFIRNYPCIRTNEGALIFVHDPATTGADKIYEGYKKFIDPH------------------------FIIYRNAEM  136
3  TVLTKTGFIRNYPCIRTNEGALIFVHDPATTGADKIYEGYKKFIDPH------------------------FIIYRNAEM  136
4  TVVTKTGLIKDYPCVRTNEGALIFVHDPTVTGADKIYEGYKKFIDPQYMTQGVSRTVKKNVKQHVPRPMNAFIIYRTAEM  151
5  TVVTKTGLIKDYPCVRTNEGALIFVHDPTVTGADKIYEGYKKFIDPQYMTQGVSRTVKKNVKQHVPRPMNAFIIYRTAEM  160

   ATVRQQYPGIGNNDVSRIIGQRWRELDLGTRNHYKVLATRSAQEHQLRHPGYKYTPRKAEDVVRRRKRSAMPSDLEHIRN
1  ATVRQQYPGIGNNDVSRIIGQRWRELDLGTRNHYKVLATRSAQEHQLRHPGYKYTPRKAEDVVRRRKRSAMPSDLEHIRN  210
2  ATVRQQYPGIGNNDVSRIIGQRWRELDLGTRNHYKVLATRSAQEHQLRHPGYKYTPRKAEDVVRRRKRSAMPSDLEHIRN  216
3  ATVRQQYPGIGNNDVSRIIGQRWRELDLGTRNHYKVLATRSAQEHQLRHPGYKYTPRKAEDVVRRRKRSAMPSDLEHIRN  216
4  VTVRQQYPGIGNNDVSRIIGQRWRELDSETRTHYKILATRSAQEHQLKHPGYKYTPRKAEDVVRRRKRSAIASDLGHIRN  231
5  VTVRQQYPGIGNNDVSRIIGQRWRELDSETRTHYKILATRSAQEHQLKHPGYKYTPRKAEDVVRRRKRSAIASDLGHIRN  240

   PKIHRGSDPIETLPVPLHNLIPGYPDLDNLTNTWVQDPTPDHFNALMDGFDSALGLAAENNLQKX
1  PKIHRGSDPIETLPVPLHNLIPGYPDLDNLTNTWVQDPTPDHFNALMDGFDSALGLAAENNLQKN  275
2  PKIHRGSDPIETLPVPLHNLIPGYPDLDNLTNTWVQDPTPDHFNALMDGFDSALGLAAENNLQKN  281
3  PKIHRGSDPIETLPVPLHNLIPGYPDLDNLTNTWVQDPTPDHFNALMDGFDSALGLAAENNLQKK  281
4  PKIHRSNDIVETPPASLHDLIPGYPDLDDLTNTWVQEPTPDHFNALMHGFDCALGLAAENNLQNI  296
5  PKIHRSNDIVETPPASLHDLIPGYPDLDDLTNTWVQEPTPDHFNALMHGFDCALGLAAENNLQNI  305
```
