# Supplementary material for: Structure of the Mating-Type Genes and Mating Systems of Verpa bohemica and Verpa conica (Ascomycota, Pezizomycotina)
Source: J Fungi (Basel). 2023 Dec 15;9(12):1202. doi: 10.3390/jof9121202 (PMC10745113; doi:10.3390/jof9121202)
Supplement: Supplementary file 1 [file jof-09-01202-s001.zip › supplementary materials/Supplementary Figure S3.pdf]

Consensus

1. MAT1-1-10 *Verpa bohemica* strain No. 20020 Gene05695
2. MAT1-1-10 *Verpa bohemica* strain No. 20124 Gene05694
3. MAT1-1-10 *Verpa bohemica* strain No. 21108 Gene05692
4. MAT1-1-10 *Verpa conica* strain No. 21110 Gene05312
5. MAT1-1-10 *Morchella sp. Mes-20* NC AVI61143
6. MAT1-1-10 *Morchella rufobrunnea* NC QQL94651

```
  MxExYLxYHDNR-RDDxRRExLLRLGHALIKVKNDLAFFPSTRLDxxIRKVLVFCIATLKEFEVKxLxxxFLDxxQDxYP
1 MTE-YLAYHDNR-RDDPRRESLLRLGHALIKVKNDLAFFPSTRLDPNIRKVLVFCIATLKEFEVKKLNLKFLDKVQDVYP 78
2 MTE-YLAYHDNR-RDDPRRESLLRLGHALIKVKNDLAFFPSTRLDPNIRKVLVFCIATLKEFEVKKLNLKFLDKVQDVYP 78
3 MTE-YLAYHDNR-RDDPRRESLLRLGHALIKVKNDLAFFPSTRLDPNIRKVLVFCIATLKEFEVKKLNLKFLDKVQDVYP 78
4 MAEAYLVCHDNR-RDDSRREALLRLGHALIKVKNDLAFFPSKRLDSQIRDVLVFCIAILKEFEVKTLRQEFLDEGQDIYP 79
5 MPQPYLSYKDHFILNDQRREAILRLGRVLIQTKNDLTFFPRTPYSFKIRKAIIFCTDTLKEFKIKNLHPALLDRHKDIYP 80
6 MPQTYLSYKDHFILNNERREAVLRLGRILIQTKNELAFFPRTPYNSKIRRVMIFCTETLKEFKIKNLHPALLDRRKDIYP 80

  LANLYNLGIQIxALxEIQxxLxTxLxQWKxDVxAVQxSFPxGSxDIGxVLAxVHEFLVFxTLTNRIHPFHTLTExSIKxA
1 LANLYNLGIQIMALLEIQDVLYTQLCQWKQDVGAVQASFPTGS-DIGIVLADVHEFLVFSTLTNRIHPFHTLTESSIKRA 157
2 LANLYNLGIQIMALLEIQDVLYTQLCQWKQDVGAVQASFPTGS-DIGIVLADVHEFLVFSTLTNRIHPFHTLTESSIKRA 157
3 LANLYNLGIQIMALLEIQNVLYTQLCQWKQDVGVVQTSFPTGS-DIGIVLADVHEFLGFSTLTNRIHPFHTLTESSIKRA 157
4 LANLYNLGIQILALQEIQNRFFTELDQWKYDVEAVQASSPGGSDDIGTVLAEVHEFLVFATLTNRIHPFHILTEFSIKHA 159
5 LANLYNLGVQILALREVNIRLERDIEKWNCGLKTLQGSLPEDSCDVRLILVEVHEFLAFATIANQIHPFHTLTPYSIRCA 160
6 LTHLYNLGVQILALREVNIRLERDIKRWNCRLKALQDSFPENSSDVGLVLAEVHEFLVFATIANQVHTSHTLTYYSIRLA 160

  VQxAKHSEFLxxASHTxSILxAAFSIQxxLxxxxTSxTKxxYVVSDAVxLxxTxIxIAxDWYKxxxxSxSxxEK------
1 VQIAKHSEFLADASHSVSILQAAFSIQSGLEALGTSNTKDKYVVSDAVTLSKTTIFIARDWYKNHELSMSAEEK------ 231
2 VQIAKHSEFLADASHSVSILQAAFSIQSGLEALGTSNTKDKYVVSDAVTLSKTTIFIARDWYKNHELSMSAEEK------ 231
3 VQIAKHSEFLADASHTVSILQAAFSIQSGLEALGASNTKDKYVVSDAVTLSQTTIFIARDWYKNHELSMSTTEK------ 231
4 VQVAKHSEFLTEASNTISILAAALSIQNELDS--ASGPKNNRVVSSAIIPPQTVIHIAKDWYKSYKSSISVAEKACCHSN 237
5 AKAAQNSRLLTEAAHTLSILRAAFSIQNELQIRRTSKTTRSRIISNAVSLPKVALNICSDWYNYRGSHA----------- 229
6 AEAAKGSIFTTEATHTISILRAAFAVQNEIQIQRTL-MRRNYIISDPVSLPQIVLTLCSDWYNHRKDHAS---------- 229

  --------xxxxIRQEAxLPxIPFxxLxTWFINARRRIxxxGQTIxPxLKxxVxxxxxSxKxPxxxxxx-xxxPMDxxxx
1 -----------QIRQEARLPVIPFDHLLTWFINARRRILVAGQTISPALKVVVHNLITSDKKPPP-----SYVPMDDSNY 295
2 -----------QIRQEARLPVIPFDHLLTWFINARRRILVAGQTISPALKVVVHNLITSDKKPPP-----SYVPMDDSNY 295
3 -----------EIRQEARLPVIPFDNLLTWFINARRRILVAGQTISPALKVVVHNLITSDKKPPP-----SHVPMDDSNY 295
4 KFHVKTANYYQEFRREAQLPMIPFNRLFTWFIN---------------------DIMAS-KVPFKHSAQ-KLTPMKNKGD 294
5 --------LYTKIRRNEISTPIPFEKLVTWFTNARRRIIKHNQAIRPGLKAIVDEALSS-KRSFKTDIH-IQTRGSNGDD 299
6 -------RIFNDFHQNDKSTQIPFENLMTWFCNGRRRIITCGQTINFDLKAIIDGAVSS-KIPSKIETRGDDTDGDNGDG 301

  ExHYxIPLTxLIAEFLDELGExPKxLLPLKHLxVDPPLPxSRSPIxFxFLVxxxADxxTQxxQxLLxxIYxLFNxxxxxC
1 EFHYNIPLTQLIAEFLDELGEVPKSLLPLKHLAVDPPLPESRSPISFDFLVDLEADRSTQIAQSLLQKIYRLFNTTIYRC 375
2 EFHYNIPLTQLIAEFLDELGEVPKSLLPLKHLAVDPPLPESRSPISFDFLVDLEADRSTQIAQSLLQKIYRLFNTTIYRC 375
3 EFHYNIPLTQLIAEFLDELGEVPKSLLPLKHLAVDPPLPESRSPISFDFLVNTEAD------------------------ 351
4 EPHYDIPLTELILELLGELGEMPKLIL-LKHSTVDPPLPRNRSPIFFNLFVNMGADGVTQETQNLLKKIYNLFNMTAYRC 373
5 DPEWEIPLSRMIAEFLDELGDAPHELDPPSNLVIN--NFGSGPPILPTLLVDNNMDKVTQGCQQLLKSIYSLFNEISCAC 377
6 EPEWEIPLSRMIAEFLDELGDAPHELDSPSHPITN--ISSRDIPLV--FPVNNSMDKFAQGCQYLLKSIYNLFNEVSSAC 377

  LxxxQxAHWLxxxxxYxYxxxAxxxxxxNxLRAxGxALATRRLNxxYFxExxVCxxxLxxxxNxNPxRSDLLEAVFEICN
1 LQVTQGAHWLSMREQYEYAVRATREIEKNYLRAVGTALATRRLNPAYFGEKIVCNRPLGAPTNNNPIRSDLLEAVFEICN 455
2 LQVTQGAHWLSMREQYEYAVRATREIEKNYLRAVGTALATRRLNPAYFGEKIVCNRPLGAPTNNNPIRSDLLEAVFEICN 455
3 -------------------------------SAVGTALATRRLNPAYFGENVVCNRPLGAPTNNNPIRSDLLEAVFEICN 400
4 LEVIQRAHWVNMEKQYEYAVRAKKEIDKNYLRAAGIALATRRLNTVHFEEEIVCRKSLVEQNNRNPFLLKVLDAVFEICN 453
5 LDIFQRAYWLDERNRYSYTLGAKRDMDYNCLRAISVLLGTRESNETYFEWQ-------------NHSRSDLFEAIFDVFP 444
6 LDILQRAHWLDAKNKQYHTSKAKIDMDRSCLRAINVLLATKKPNKTYLECQ-------------NHPWSDLLEKIFEVFP 444
```

```
  DPNILEEEYLXXITGLS--VXQXHXWX-----------LYXXXYXXXXXXXXXXXI
1 DPNILEEEYLTSITGLS--VTQVHCWL----------VLYKRHYLSQPTMVDVDEI  499
2 DPNILEEEYLTSITGLS--VTQVHCWF-----------LYKRHYLSQPTMVDVDEI  498
3 DPNILEEEYLTSITGLS--VTQVHCWF-----------LYKRHYLSQPTMADVDEI  443
4 DPNMLEEEYLAYVTGLSFFINAIN--------------------------------  477
5 NPNITEEEYLSKITGMN--VNQIHTWCEKSFNH-----------------------  475
6 DPNTAEEEYMSYATGMN--INQIHTSCKGSINLNSVNCLYFNIYV----------I  488
```
