# Supplementary material for: Structure of the Mating-Type Genes and Mating Systems of Verpa bohemica and Verpa conica (Ascomycota, Pezizomycotina)
Source: J Fungi (Basel). 2023 Dec 15;9(12):1202. doi: 10.3390/jof9121202 (PMC10745113; doi:10.3390/jof9121202)
Supplement: Supplementary file 1 [file jof-09-01202-s001.zip › supplementary materials/Supplementary Figure S4.pdf]

Consensus

1. MAT1-1-11 *Verpa conica* strain No. 21117 Gene05376
2. MAT1-1-11 *Verpa conica* strain No. 21120 Gene05383
3. MAT1-1-11 *Morchella importuna* NC AVI61142

```
  --------------------MNS-------LLNEGKIRGSYSKIFSLLLTFIVANVSNRRKAIDRVVETLSQIRNELAYK
1 --------------------MNS-------LLNEGKIRGSYSKIFSLLLTFIVANVSNRRKAIDRVVETLSQIRNELAYK 53
2 MIDFHLHSEHSRTVIPPINDMNS-------LLNEGKIRGSYSKIFSLLLTFIVANVSNRRKAIDRVVETLSQIRNELAYK 73
3 --------------------MDAVNCQPVVVANEA--------------TITPRKITSRREAINKIRNVLCQVKNELAYK 46

  PGAITDAKIRDVLLFALRVVRGFRVKYLRIKNLDETLDFYPLVNIHNLGRQIFSLFELQKKFTKSLRDVAIELKILQTSI
1 PGAITDAKIRDVLLFALRVVRGFRVKYLRIKNLDETLDFYPLVNIHNLGRQIFSLFELQKKFTKSLRDVAIELKILQTSI 133
2 PGAITDAKIRDVLLFALRVVRGFRVKYLRIKNLDETLDFYPLVNIHNLGRQIFSLFELQKKFTKSLRDVAIELKILQTSI 153
3 PRAVPDAKVRDVAIFSTRVLRRFRIKELKRDSIDERLDFHPIADIYSLGHQVKSLLGAQSNLVRDLRYLEEKIQKLQHNY 126

  FKGEENIRVAIAEAHEFLLFATFINQLHRDDFFDDDTLQCATSHLEDSPHVEAAHALSGLRVALSVSKELEKKDCREX-P
1 FKGEENIRVAIAEAHEFLLFATFINQLHRDDFFDDDTLQCATSHLEDSPHVEAAHALSGLRVALSVSKELEKKDCRET-P 212
2 FKGEENIRVAIAEAHEFLLFATFINQLHRDDFFDDDTLQCATSHLEDSPHVEAAHALSGLRVALSVSKELEKKDCREN-P 232
3 ---TEDIWVAIADLHEFLIFACFVNQIHRDDTLDEKTLYSARNHLADSPHPEAGIALADLRAARQVEKEFQKMNHGEGLP 203

  TVSSPTKLSPITLSIAREWYKNYEINMANDEREWRKLCFFPLTPFDTMLDWFTKARCRINSDTRAIRPAFKVIVDKAMKA
1 TVSSPTKLSPITLSIAREWYKNYEINMANDEREWRKLCFFPLTPFDTMLDWFTKARCRINSDTRAIRPAFKVIVDKAMKA 292
2 TVSSPTKLSPITLSIAREWYKNYEINMANDEREWRKLCFFPLTPFDTMLDWFTKARCRINSDTRAIRPAFKVIVDKAMKA 312
3 TVSKVIKLGPLATSLASEWYRNYELHMPANEQEWRNLCRFPLPSFETMFGWFENSRRRILSDIHSIRPGLKAIIDKQIRD 283

  KEVLAGFQKGQVKDDTRRDIEPINDLLKALILEVEGFGSLVKIRPFSPPPFPP-SRAPENKSPINLEYRISD--SINPSI
1 KEVLAGFQKGQVKDDTRRDIEPINDLLKALILEVEGFGSLVKIRPFSPPPFPP-SRAPENKSPINLEYRISD--SINPSI 369
2 KEVLAGFQKGQVKDDTRRDIEPINDLLKALILEVEGFGSLVKIRPFSPPPFPP-SRAPENKSPINLEYRISD--SINPSI 389
3 DSVVTKLNKGISMDEKRGETEKLDTLLADMIHEVDKLGSQLKMKAFTPPSFPPASCTPQNHSPIKLQYWVSDTCTIDGNM 363

  QEIQRLIKVIFESFATTAALSMKIILRDHGLDEVQEITSSQHSEREFNTRYL----------------------------
1 QEIQRLIKVIFESFATTAALSMKIILRDHGLDEVQEITSSQHSEREFNTRYL---------------------------- 421
2 QEIQRLIKVIFESFATTAALSMKIILRDHGLDEVQEITSSQHSEREFNTRYL---------------------------- 441
3 KQIQHIIGRIFEAFTGTADFCMNAILHSDRLGGYEEKVFAQKSEKEFNIRYLSAVGEFLGQMRFHQVFEAINPDEFGSKE 443

  ------------RYNVPVLNIV--------------------------------------------SE
1 ------------RYNVPVLNIV--------------------------------------------SE 433
2 ------------RYNVPVLNIV--------------------------------------------SE 453
3 LTPETRNLILRDRHHSQILTCIFDAFGSLSEAEEEYLLGITHLHMSLEEIHEWFQKHSAKFQNDKLEK 511
```
