# Supplementary material for: Structure of the Mating-Type Genes and Mating Systems of Verpa bohemica and Verpa conica (Ascomycota, Pezizomycotina)
Source: J Fungi (Basel). 2023 Dec 15;9(12):1202. doi: 10.3390/jof9121202 (PMC10745113; doi:10.3390/jof9121202)
Supplement: Supplementary file 1 [file jof-09-01202-s001.zip › supplementary materials/Supplementary Figure S5.pdf]

The detailed sequence information of the *MAT1-1-10* protein motif.

Motif 1

Name	Start	*p*-value		Sites	
Morchella_purpurascens_[QQL94643.1]	48	1.27e-60	FFPRTPYNPK	IRKVMVFCIATLKEFKIKNLHPALLDQHKDIYPLAHLYNLGVQILALREV	NIRLEKDIIK
Morchella_importuna_[AVI60817.1]	48	1.42e-58	FFPRTAYNLK	IRKAMVFCIATLKEFKIKNLHPALLDQHKDICPLAHLYNLGVQILALREV	NIRLEKDIIK
Morchella_sextelata_[QQL94615.1]	48	3.25e-58	FFPRTPYNPK	IRKAMVFCIAILKEFKIKNLHPALLDQHKDIYPLAHLYNLGVQILALREV	NIRLEKDIIK
Morchella_sp._Mes-20_[AVI61143.1]	48	1.75e-57	FFPRTPYSFK	IRKAIIFCTDTLKEFKIKNLHPALLDRHKDIYPLANLYNLGVQILALREV	NIRLERDIEK
Morchella_sp._Mes-19_[QQL94629.1]	48	8.09e-56	FFPRTPYSFK	IRKAIIFCTDTLKELKIKNLHPALLDRHKDIYPLANLYNLGVQILALREV	NIRLERDIEK
Morchella_crassipes_[AVI60838.1]	48	8.09e-56	FFPRTPYSFK	IRKAIIFCTDTLKELKIKNLHPALLDRHKDIYPLANLYNLGVQILALREV	NIRLERDIEK
Morchella_sp._Mes-6_[QQL94636.1]	48	4.76e-55	FFPRTPYSFK	IRKAIRFCTDTLKELKIKNLHPALLDRHKDIYPLANLYNLGVQILALREV	NIRLERDIEK
Morchella_rufobrunnea_[QQL94651.1]	48	2.94e-53	FFPRTPYNSK	IRRVMIFCTETLKEFKIKNLHPALLDRRKDIYPLTHLYNLGVQILALREV	NIRLERDIKR
V._bohemica_No._21108	46	1.76e-50	FFPSTRLDPN	IRKVLVFCIATLKEFEVKKLNLKFLDKVQDVYPLANLYNLGIQIMALLEI	QNVLYTQLCQ
V._bohemica_No._21124	46	1.76e-50	FFPSTRLDPN	IRKVLVFCIATLKEFEVKKLNLKFLDKVQDVYPLANLYNLGIQIMALLEI	QDVLYTQLCQ
V._bohemica_No._20020	46	1.76e-50	FFPSTRLDPN	IRKVLVFCIATLKEFEVKKLNLKFLDKVQDVYPLANLYNLGIQIMALLEI	QDVLYTQLCQ
V._conica_No._21110	47	2.92e-45	FFPSKRLDSQ	IRDVLVFCIAILKEFEVKTLRQEFLDEGQDIYPLANLYNLGIQILALQEI	QNRFFTELDQ

Motif 2

Name	Start	*p*-value		Sites	
Morchella_crassipes_[AVI60838.1]	118	3.16e-59	WNCGLKTLQS	SLPEDSCDVGLILVEVHEFLVFATIANQIHPFHTLTPYSIRCAAKAAQNS	RLLTEAAHTL
Morchella_sp._Mes-6_[QQL94636.1]	118	3.16e-59	WNCGLKTLQG	SLPEDSCDVGLILVEVHEFLVFATIANQIHPFHTLTPYSIRCAAKAAQNS	RLLTEAAHTI
Morchella_sp._Mes-19_[QQL94629.1]	118	3.78e-58	WNCGLKTLQG	SLPGDSCDVGLILVEVHEFLVFATIANQIHPFHTLTPYSIRCAAKAAQDS	RLLTEAAHTL
Morchella_sp._Mes-20_[AVI61143.1]	118	1.10e-54	WNCGLKTLQG	SLPEDSCDVRLILVEVHEFLAFATIANQIHPFHTLTPYSIRCAAKAAQNS	RLLTEAAHTL
Morchella_purpurascens_[QQL94643.1]	118	3.56e-54	WDRNLKALQD	SLPEYSEDVGLILAEVHEFLAFATVANQIHPFDTLTSSSIKCAAIAAKNS	GSPIEDSHAI
Morchella_importuna_[AVI60817.1]	118	1.24e-53	WDHSLKTLQD	SLPEHSEDVGLILAEVHEFLAFATVANQIHPFDTLTSSSIKCAAIAAKDS	GSLIEDSHAI
Morchella_sextelata_[QQL94615.1]	118	5.70e-52	WDHSLKALQD	SLPEYSEDVGLILAEVHEFLGFATVANQIHPFDTLTSSSIKRAATAANDS	GSLIEDSHAI
V._bohemica_No._21124	115	9.02e-49	QWKQDVGAVQ	ASFPTGSDIGIVLADVHEFLVFSTLTNRIHPFHTLTESSIKRAVQIAKHS	EFLADASHSV
V._bohemica_No._20020	115	9.02e-49	QWKQDVGAVQ	ASFPTGSDIGIVLADVHEFLVFSTLTNRIHPFHTLTESSIKRAVQIAKHS	EFLADASHSV
V._bohemica_No._21108	115	1.60e-48	QWKQDVGVVQ	TSFPTGSDIGIVLADVHEFLGFSTLTNRIHPFHTLTESSIKRAVQIAKHS	EFLADASHTV
V._conica_No._21110	117	2.12e-48	WKYDVEAVQA	SSPGGSDDIGTVLAEVHEFLVFATLTNRIHPFHILTEFSIKHAVQVAKHS	EFLTEASNTI
Morchella_rufobrunnea_[QQL94651.1]	118	8.59e-48	WNCRLKALQD	SFPENSSDVGLVLAEVHEFLVFATIANQVHTSHTLTYYSIRLAAEAAKGS	IFTTEATHTI

Motif 3

Name	Start	*p*-value		Sites	
Morchella_sp._Mes-19_[QQL94629.1]	232	2.66e-56	YNYRGSHTLY	TKIRRNDISTPIPFEKLVTWFTNARRRIIKHNQAIRPGLKAIVDEALSSK	RSFKTDIYIQ
Morchella_sp._Mes-20_[AVI61143.1]	232	1.58e-55	YNYRGSHALY	TKIRRNEISTPIPFEKLVTWFTNARRRIIKHNQAIRPGLKAIVDEALSSK	RSFKTDIHIQ
Morchella_crassipes_[AVI60838.1]	232	1.34e-54	YNYRGSHALY	TKIRRNDISTPIPFEKLVTWFTNARRRIIRRGQAIRPGLKAIVDEALSSK	RSFKTDIHIQ
Morchella_importuna_[AVI60817.1]	232	4.97e-54	YNCYKSYASC	TGIHQNTISTMIPFENLVTWFTNARRRIIGGDQSISPGLKSIVNGALSSK	PLKIDHHVQH
Morchella_sp._Mes-6_[QQL94636.1]	234	3.63e-53	YNYRGSHALY	TKIRRNDISTPIPFENLVTWFTNARRRILKHNQAIRPSLKAIVDEALSSK	RSFKTDIHIQ
Morchella_sextelata_[QQL94615.1]	234	6.09e-52	CYKSHAPCTV	NEIHQNTISTMIPFENLVTWFTNARRRIIGGNQTISPGLESIVNGALSSK	SLKIDHHVQD
V._bohemica_No._21108	231	1.95e-45	NHELSMSTTE	KEIRQEARLPVIPFDNLLTWFINARRRILVAGQTISPALKVVVHNLITSD	KKPPPSHVPM
V._bohemica_No._21124	231	2.64e-45	NHELSMSAEE	KQIRQEARLPVIPFDHLLTWFINARRRILVAGQTISPALKVVVHNLITSD	KKPPPSYVPM
V._bohemica_No._20020	231	2.64e-45	NHELSMSAEE	KQIRQEARLPVIPFDHLLTWFINARRRILVAGQTISPALKVVVHNLITSD	KKPPPSYVPM
Morchella_purpurascens_[QQL94643.1]	230	1.72e-44	SWYNCYKSYT	SCTVNNTISTVIRFENLVTWFTNARRRLIGGDQTISPGLKSIVNEALSKP	SKIDCHVQDK
Morchella_rufobrunnea_[QQL94651.1]	233	2.08e-43	HRKDHASRIF	NDFHQNDKSTQIPFENLMTWFCNGRRRIITCGQTINFDLKAIIDGAVSSK	IPSKIETRGD

Motif 4

Name	Start	*p*-value		Sites	
Morchella_sp._Mes-6_[QQL94636.1]	425	1.40e-59	VLLATREFNE	TYFEWQNHSRSDLFEAIFDVFPNPNITEEEYLSKITGMNVNQIHTWCEKS	FNR
Morchella_sp._Mes-20_[AVI61143.1]	423	1.40e-59	VLLGTRESNE	TYFEWQNHSRSDLFEAIFDVFPNPNITEEEYLSKITGMNVNQIHTWCEKS	FNH
Morchella_sp._Mes-19_[QQL94629.1]	423	9.79e-57	VLLATREPNE	TYFEWQNHSRSDLFEAIFDVFPNPNITEEEYLSKITGINVNQIHTWCEKS	FNH
Morchella_purpurascens_[QQL94643.1]	417	8.69e-56	ILLATRGPNQ	NYFEWKRHSRSELLEAIFEIYPDPNMVEEEYLSNVTGMNINQIHTWCKRS	LDPNPYTRIH
Morchella_sextelata_[QQL94615.1]	422	2.58e-54	ILLATRGANQ	NHFEWKRHSRSELLETIFETFPDPNMIEEEYLSNVTGMNINQVHTWCKKS	LDPQSYLYTL
Morchella_importuna_[AVI60817.1]	420	6.64e-51	ILLATRGLNQ	DHFERKRHSRPELLETIFEIFPDPNLMEEEYLSNVTGMNINQIHTWCRKS	PDPQSYLYTL
V._bohemica_No._21108	379	3.53e-47	GENVVCNRPL	GAPTNNNPIRSDLLEAVFEICNDPNILEEEYLTSITGLSVTQVHCWFLYK	RHYLSQPTMA
V._bohemica_No._21124	434	3.53e-47	GEKIVCNRPL	GAPTNNNPIRSDLLEAVFEICNDPNILEEEYLTSITGLSVTQVHCWFLYK	RHYLSQPTMV
Morchella_rufobrunnea_[QQL94651.1]	423	5.01e-46	VLLATKKPNK	TYLECQNHPWSDLLEKIFEVFPDPNTAEEEYMSYATGMNINQIHTSCKGS	INLNSVNCLY
V._bohemica_No._20020	434	1.08e-45	GEKIVCNRPL	GAPTNNNPIRSDLLEAVFEICNDPNILEEEYLTSITGLSVTQVHCWLVLY	KRHYLSQPTM

Motif 5

Name	Start	*p*-value		Sites	
Morchella_sp._Mes-6_[QQL94636.1]	342	8.58e-60	NFVINNFGSG	PPILPTFLVDNNMDKVTQGCQHLLKSIYSLFNEISCACLDIFQRAYWLDE	RNRCSYTLGA
Morchella_importuna_[AVI60817.1]	338	6.38e-57	HIIINRFDIE	PPINPMFFANNSADKLTQGCQHLLKSIYSLFNEISSACLDIFQRAHWLNK	RRYDYALKAK
Morchella_sp._Mes-20_[AVI61143.1]	340	2.62e-55	NLVINNFGSG	PPILPTLLVDNNMDKVTQGCQQLLKSIYSLFNEISCACLDIFQRAYWLDE	RNRYSYTLGA
Morchella_sextelata_[QQL94615.1]	340	3.69e-54	HIIINHFDIE	PPINPLFFVNNSADKLTQGCLHLLKSIYNLFNEISSACLDIFQRAHWLNK	RRYDYALKAK
Morchella_purpurascens_[QQL94643.1]	335	4.76e-54	HIIINHFNIE	PRINPMFFVNITTDKLTQGCQHLLKSIYNLFNEISSACLDIFQRAHWLNK	RRYDYALKAK
Morchella_sp._Mes-19_[QQL94629.1]	340	7.91e-54	NFVINNFGSG	PPILPTFLVDNNIDKVTQGCQRQLKSIYSLFNEISCACLDIFQRAYWLDE	RNRYSYTLGA
Morchella_rufobrunnea_[QQL94651.1]	340	3.25e-46	PSHPITNISS	RDIPLVFPVNNSMDKFAQGCQYLLKSIYNLFNEVSSACLDILQRAHWLDA	KNKQYHTSKA
V._bohemica_No._21124	338	5.24e-42	AVDPPLPESR	SPISFDFLVDLEADRSTQIAQSLLQKIYRLFNTTIYRCLQVTQGAHWLSM	REQYEYAVRA
V._bohemica_No._20020	338	5.24e-42	AVDPPLPESR	SPISFDFLVDLEADRSTQIAQSLLQKIYRLFNTTIYRCLQVTQGAHWLSM	REQYEYAVRA
V._conica_No._21110	336	7.41e-42	TVDPPLPRNR	SPIFFNLFVNMGADGVTQETQNLLKKIYNLFNMTAYRCLEVIQRAHWVNM	EKQYEYAVRA

Motif 6

Name	Start	*p*-value	Sites
Morchella_sp._Mes-19_[QQL94629.1]	13	1.23e-36	QPYLPYKDHF ILNDQRREAILRLGRVLIQTKNDLTFFPRTP YSFKIRKAII
Morchella_crassipes_[AVI60838.1]	13	1.23e-36	QPYLPYKDHF ILNDQRREAILRLGRVLIQTKNDLTFFPRTP YSFKIRKAII
Morchella_sp._Mes-6_[QQL94636.1]	13	1.23e-36	QPYLSYEDHF ILNDQRREAILRLGRVLIQTKNDLTFFPRTP YSFKIRKAIR
Morchella_sp._Mes-20_[AVI61143.1]	13	1.23e-36	QPYLSYKDHF ILNDQRREAILRLGRVLIQTKNDLTFFPRTP YSFKIRKAII
Morchella_sextelata_[QQL94615.1]	13	3.74e-34	QPYLSYDDHF LLNHERREAILRLGQTLIQTKNDLAFFPRTP YNPKIRKAMV
Morchella_purpurascens_[QQL94643.1]	13	6.94e-33	QQYLSYHDHF LLDHERREAILRLGQTLIQMKNDLVFFPRTP YNPKIRKVMV
Morchella_importuna_[AVI60817.1]	13	1.26e-32	QPYLSYDDHF LLNHERREAILRLGQTLIQTKNDLTFFPRTA YNLKIRKAMV
V._bohemica_No._21108	11	1.83e-31	MTEYLAYHDN RRDDPRRESLLRLGHALIKVKNDLAFFPSTR LDPNIRKVLV
V._bohemica_No._21124	11	1.83e-31	MTEYLAYHDN RRDDPRRESLLRLGHALIKVKNDLAFFPSTR LDPNIRKVLV
V._bohemica_No._20020	11	1.83e-31	MTEYLAYHDN RRDDPRRESLLRLGHALIKVKNDLAFFPSTR LDPNIRKVLV
Morchella_rufobrunnea_[QQL94651.1]	13	4.59e-31	QTYLSYKDHF ILNNERREAVLRLGRILIQTKNELAFFPRTP YNSKIRRVMI
V._conica_No._21110	12	3.37e-30	AEAYLVCHDN RRDDSRREALLRLGHALIKVKNDLAFFPSKR LDSQIRDVLV

Motif 7

Name	Start	*p*-value	Sites
Morchella_sp._Mes-20_[AVI61143.1]	289	2.07e-48	SSKRSFKTDI HIQTRGSNGDDDPEWEIPLSRMIAEFLDELGDAPHELDPPS NLVINNFGSG
Morchella_sp._Mes-6_[QQL94636.1]	291	7.69e-46	SSKRSFKTDI HIQTRGSNGDDDPEWETPLSRMIAEFLDELGDAPHELDPPS NFVINNFGSG
Morchella_crassipes_[AVI60838.1]	289	4.83e-45	SSKRSFKTDI HIQARGSNGDDDPEWEIPLSRAIAEFLDELGDAPHELDPPS NFVINNFGSG
Morchella_sextelata_[QQL94615.1]	290	5.60e-45	LSSKSLKIDH HVQDKSDNGDGDPEWEIPLSRLIAEFLDELGDAPKFVSPNH IIINHFDIEP
Morchella_sp._Mes-19_[QQL94629.1]	289	2.54e-43	SSKRSFKTDI YIQARGSNGDDDPEWEIPLSRTIAEFLDELGDAPHELDPPS NFVINNFGSG
Morchella_importuna_[AVI60817.1]	288	2.89e-43	LSSKPLKIDH HVQHKSDNGDGDPEWEIPLSRLIAEFLDELGDTPKFVSPNH IIINRFDIEP
Morchella_purpurascens_[QQL94643.1]	285	3.10e-41	ALSKPSKIDC HVQDKSDNGDGDPEWEVPLSRLIAEFLDELGDAPGFVSFDH IIINHFNIEP
Morchella_rufobrunnea_[QQL94651.1]	291	3.15e-40	SKIPSKIETR GDDTDGDNGDGEPEWEIPLSRMIAEFLDELGDAPHELDSPS HPITNISSRD
V._bohemica_No._21124	285	7.66e-35	NLITSDKKPP PSYVPMDDSNYEFHYNIPLTQLIAEFLDELGEVPKSLLPLK HLAVDPPLPE
V._bohemica_No._20020	285	7.66e-35	NLITSDKKPP PSYVPMDDSNYEFHYNIPLTQLIAEFLDELGEVPKSLLPLK HLAVDPPLPE
V._bohemica_No._21108	285	8.35e-35	NLITSDKKPP PSHVPMDDSNYEFHYNIPLTQLIAEFLDELGEVPKSLLPLK HLAVDPPLPE
V._conica_No._21110	284	1.14e-26	ASKVPFKHSA QKLTPMKNKGDEPHYDIPLTELILELLGELGEMPKLILLKH STVDPPLPRN

Motif 8

Name	Start	*p*-value	Sites
Morchella_crassipes_[AVI60838.1]	201	5.56e-32	QVRRTSKTTR SRIISNAVSLPKVALNICSDWYNYRGSHA LYTKIRRNDI
Morchella_sp._Mes-20_[AVI61143.1]	201	5.56e-32	QIRRTSKTTR SRIISNAVSLPKVALNICSDWYNYRGSHA LYTKIRRNEI
Morchella_importuna_[AVI60817.1]	201	7.76e-32	QIQRTSNSGI NHVITNAVSLPQIAIVICSDWYNCYKSYA SCTGIHQNTI
Morchella_sp._Mes-19_[QQL94629.1]	201	2.77e-31	QIRRMSKTTR SRIISNAVSLPKVALNICSDWYNYRGSHT LYTKIRRNDI
Morchella_sextelata_[QQL94615.1]	201	2.48e-30	QIQRTSNSGI NHVITDAVSLPQIAIVICSNWYNCYKSHA PCTVNEIHQN
Morchella_sp._Mes-6_[QQL94636.1]	203	1.53e-28	RRTSKTTRSR IIMISNAVSLQKVALNICSDWYNYRGSHA LYTKIRRNDI
V._bohemica_No._21108	198	1.26e-26	EALGASNTKD KYVVSDAVTLSQTTIFIARDWYKNHELSM STTEKEIRQE
V._bohemica_No._21124	198	1.55e-26	EALGTSNTKD KYVVSDAVTLSKTTIFIARDWYKNHELSM SAEEKQIRQE
V._bohemica_No._20020	198	1.55e-26	EALGTSNTKD KYVVSDAVTLSKTTIFIARDWYKNHELSM SAEEKQIRQE
Morchella_rufobrunnea_[QQL94651.1]	200	6.03e-26	IQIQRTLMRR NYIISDPVSLPQIVLTLCSDWYNHRKDHA SRIFNDFHQN
Morchella_purpurascens_[QQL94643.1]	201	2.24e-25	HIQRTSNSGI NHVITNVVPLPQIAISICSSWYNCYKSYT SCTVNNTIST
V._conica_No._21110	198	4.93e-22	ELDSASGPKN NRVVSSAIIPPQTVIHIAKDWYKSYKSSI SVAEKACCHS

Motif 9

Name	Start	*p*-value	Sites
Morchella_sp._Mes-6_[QQL94636.1]	170	2.05e-33	AAKAAQNSRL LTEAAHTISILRAAFSIQNELQIRRTSKT TRSRIIMISN
Morchella_sp._Mes-20_[AVI61143.1]	170	5.45e-32	AAKAAQNSRL LTEAAHTLSILRAAFSIQNELQIRRTSKT TRSRIISNAV
Morchella_sp._Mes-19_[QQL94629.1]	170	3.30e-31	AAKAAQDSRL LTEAAHTLSILRAAFSIQNELQIRRMSKT TRSRIISNAV
Morchella_crassipes_[AVI60838.1]	170	3.30e-31	AAKAAQNSRL LTEAAHTLSILRAAFSIQNELQVRRTSKT TRSRIISNAV
Morchella_sextelata_[QQL94615.1]	170	1.77e-29	AATAANDSGS LIEDSHAISILQAAFTIQTELQIQRTSNS GINHVITDAV
Morchella_importuna_[AVI60817.1]	170	1.98e-29	AAIAAKDSGS LIEDSHAISILQAAFTIQTGLQIQRTSNS GINHVITNAV
V._bohemica_No._21124	167	7.68e-28	AVQIAKHSEF LADASHSVSILQAAFSIQSGLEALGTSNT KDKYVVSDAV
V._bohemica_No._20020	167	7.68e-28	AVQIAKHSEF LADASHSVSILQAAFSIQSGLEALGTSNT KDKYVVSDAV
V._bohemica_No._21108	167	2.62e-27	AVQIAKHSEF LADASHTVSILQAAFSIQSGLEALGASNT KDKYVVSDAV
Morchella_purpurascens_[QQL94643.1]	170	7.74e-26	AAIAAKNSGS PIEDSHAISILQAAFTIQTALHIQRTSNS GINHVITNVV
Morchella_rufobrunnea_[QQL94651.1]	170	3.37e-24	AAEAAKGSIF TTEATHTISILRAAFAVQNEIQIQRTLMR RNYIISDPVS
V._conica_No._21110	169	8.81e-21	AVQVAKHSEF LTEASNTISILAAALSIQNELDSASGPKN NRVVSSAIIP

Motif 10

Name	Start	*p*-value	Sites		
Morchella_sp._Mes-19_[QQL94629.1]	393	3.91e-34	RAYWLDERNR	YSYTLGAKRDMDYNCLRAISVLLATREPN	ETYFEWQNHS
Morchella_purpurascens_[QQL94643.1]	387	1.27e-32	QRAHWLNKRR	YDYALKAKRDIDDNCLRAITILLATRGPN	QNYFEWKRHS
Morchella_importuna_[AVI60817.1]	390	5.42e-32	QRAHWLNKRR	YDYALKAKRDIDDNCLRAITILLATRGLN	QDHFERKRHS
Morchella_sp._Mes-6_[QQL94636.1]	395	1.74e-31	RAYWLDERNR	CSYTLGAKRDMDYNCLRAISVLLATREFN	ETYFEWQNHS
Morchella_sextelata_[QQL94615.1]	392	1.74e-31	QRAHWLNKRR	YDYALKAKRDIDDNCLRAITILLATRGAN	QNHFEWKRHS
Morchella_sp._Mes-20_[AVI61143.1]	393	2.52e-31	RAYWLDERNR	YSYTLGAKRDMDYNCLRAISVLLGTRESN	ETYFEWQNHS
V._conica_No._21110	389	8.00e-27	RAHWVNMEKQ	YEYAVRAKKEIDKNYLRAAGIALATRRLN	TVHFEEEIVC
V._bohemica_No._21124	391	2.10e-26	GAHWLSMREQ	YEYAVRATREIEKNYLRAVGTALATRRLN	PAYFGEKIVC
V._bohemica_No._20020	391	2.10e-26	GAHWLSMREQ	YEYAVRATREIEKNYLRAVGTALATRRLN	PAYFGEKIVC
Morchella_rufobrunnea_[QQL94651.1]	393	4.59e-23	RAHWLDAKNK	QYHTSKAKIDMDRSCLRAINVLLATKKPN	KTYLECQNHP

Motif 11

Name	Start	*p*-value	Sites		
Morchella_sp._Mes-19_[QQL94629.1]	102	1.71e-18	LALREVNIRL	ERDIEKWNCGLKTLQ	GSLPGDSCDV
Morchella_crassipes_[AVI60838.1]	102	1.71e-18	LALREVNIRL	ERDIEKWNCGLKTLQ	SSLPEDSCDV
Morchella_sp._Mes-6_[QQL94636.1]	102	1.71e-18	LALREVNIRL	ERDIEKWNCGLKTLQ	GSLPEDSCDV
Morchella_sp._Mes-20_[AVI61143.1]	102	1.71e-18	LALREVNIRL	ERDIEKWNCGLKTLQ	GSLPEDSCDV
V._bohemica_No._21124	100	3.09e-15	MALLEIQDVL	YTQLCQWKQDVGAVQ	ASFPTGSDIG
V._bohemica_No._20020	100	3.09e-15	MALLEIQDVL	YTQLCQWKQDVGAVQ	ASFPTGSDIG
Morchella_sextelata_[QQL94615.1]	102	3.86e-15	LALREVNIRL	EKDIIKWDHSLKALQ	DSLPEYSEDV
Morchella_importuna_[AVI60817.1]	102	4.31e-15	LALREVNIRL	EKDIIKWDHSLKTLQ	DSLPEHSEDV
Morchella_rufobrunnea_[QQL94651.1]	102	6.66e-15	LALREVNIRL	ERDIKRWNCRLKALQ	DSFPENSSDV
Morchella_purpurascens_[QQL94643.1]	102	1.69e-14	LALREVNIRL	EKDIIKWDRNLKALQ	DSLPEYSEDV
V._bohemica_No._21108	100	1.69e-14	MALLEIQNVL	YTQLCQWKQDVGVVQ	TSFPTGSDIG
V._conica_No._21110	101	2.28e-12	LALQEIQNRF	FTELDQWKYDVEAVQ	ASSPGGSDDI

Motif 12

Name	Start	*p*-value	Sites		
Morchella_sp._Mes-20_[AVI61143.1]	1	1.57e-15		MPQPYLSYKDH	FILNDQRREA
Morchella_importuna_[AVI60817.1]	1	6.45e-15		MPQPYLSYDDH	FLLNHERREA
Morchella_sp._Mes-19_[QQL94629.1]	1	8.04e-15		MPQPYLPYKDH	FILNDQRREA
Morchella_crassipes_[AVI60838.1]	1	8.04e-15		MPQPYLPYKDH	FILNDQRREA
Morchella_sp._Mes-6_[QQL94636.1]	1	9.45e-15		MPQPYLSYEDH	FILNDQRREA
Morchella_sextelata_[QQL94615.1]	1	4.32e-14		MSQPYLSYDDH	FLLNHERREA
Morchella_rufobrunnea_[QQL94651.1]	1	1.36e-13		MPQTYLSYKDH	FILNNERREA
Morchella_purpurascens_[QQL94643.1]	1	1.36e-13		MPQQYLSYHDH	FLLDHERREA
V._bohemica_No._21124	487	2.07e-10	HCWFLYKRHY	LSQPTMVDVDE	I
V._bohemica_No._20020	488	2.07e-10	CWLVLYKRHY	LSQPTMVDVDE	I
V._bohemica_No._21108	432	5.37e-10	HCWFLYKRHY	LSQPTMADVDE	I
V._conica_No._21110	1	6.62e-9		MAEAYLVCHDN	RRDDSRREAL

Motif 13

Name	Start	*p*-value	Sites		
Morchella_sp._Mes-19_[QQL94629.1]	332	4.85e-11	PHELDPPSNF	VINNFGSG	PPILPTFLVD
Morchella_crassipes_[AVI60838.1]	332	4.85e-11	PHELDPPSNF	VINNFGSG	PPILPTFLVD
Morchella_sp._Mes-6_[QQL94636.1]	334	4.85e-11	PHELDPPSNF	VINNFGSG	PPILPTFLVD
Morchella_sp._Mes-20_[AVI61143.1]	332	4.85e-11	PHELDPPSNL	VINNFGSG	PPILPTLLVD

Motif 14

Name	Start	*p*-value	Sites		
V._bohemica_No._21124	422	1.86e-15	ALATRRLNPA	YFGEKIVCNRP	LGAPTNNNPI
V._bohemica_No._20020	422	1.86e-15	ALATRRLNPA	YFGEKIVCNRP	LGAPTNNNPI
V._bohemica_No._21108	367	1.40e-14	ALATRRLNPA	YFGENVVCNRP	LGAPTNNNPI

Motif 15

Name	Start	p-value		Sites	
V._bohemica_No._21108	1	1.09e-11		MTEYLAYH	DNRRDDPRRE
V._bohemica_No._21124	1	1.09e-11		MTEYLAYH	DNRRDDPRRE
V._bohemica_No._20020	1	1.09e-11		MTEYLAYH	DNRRDDPRRE
V._conica_No._21110	459	2.81e-8	FEICNDPNML	EEEYLAYV	TGLSFFINAI

Motif 16

Name	Start	p-value		Sites	
V._bohemica_No._21108	328	5.95e-13	PKSLLPLKHL	AVDPPLPESR	SPISFDFLVN
V._bohemica_No._21124	328	5.95e-13	PKSLLPLKHL	AVDPPLPESR	SPISFDFLVD
V._bohemica_No._20020	328	5.95e-13	PKSLLPLKHL	AVDPPLPESR	SPISFDFLVD
V._conica_No._21110	326	2.25e-10	MPKLILLKHS	TVDPPLPRNR	SPIFFNLFVN

Motif 17

Name	Start	p-value		Sites	
Morchella_sp._Mes-19_[QQL94629.1]	282	3.03e-8	AIVDEALSSK	RSFKTD	IYIQARGSNG
Morchella_crassipes_[AVI60838.1]	282	3.03e-8	AIVDEALSSK	RSFKTD	IHIQARGSNG
Morchella_sp._Mes-6_[QQL94636.1]	284	3.03e-8	AIVDEALSSK	RSFKTD	IHIQTRGSNG
Morchella_sp._Mes-20_[AVI61143.1]	282	3.03e-8	AIVDEALSSK	RSFKTD	IHIQTRGSNG
