# Supplementary material for: Structure of the Mating-Type Genes and Mating Systems of Verpa bohemica and Verpa conica (Ascomycota, Pezizomycotina)
Source: J Fungi (Basel). 2023 Dec 15;9(12):1202. doi: 10.3390/jof9121202 (PMC10745113; doi:10.3390/jof9121202)
Supplement: Supplementary file 1 [file jof-09-01202-s001.zip › supplementary materials/Supplementary Figure S6.pdf]

The detailed sequence information of the *MAT1-1-11* protein motif.

## Motif 1

| Name | Start | *p*-value | Sites |
|---|---|---|---|
| Morchella_purpurascens_[QQL94647] | 222 | 2.01e-61 | GPLATSLASE WYRNYKLHMPADEQEWRNSCRFPLPPFETMLGWFENSRRRILSDIHSIRP GFKAIIDKQI |
| Morchella_sextelata_[QQL94614] | 222 | 2.33e-61 | GPLATSLATE WYRNYELHMPADGQEWRNLCRFPLPSFETMLGWFENSRRRILSDIHSIRP GLKAIIDKQI |
| Morchella_importuna_[AVI61142] | 222 | 1.28e-60 | GPLATSLASE WYRNYELHMPANEQEWRNLCRFPLPSFETMFGWFENSRRRILSDIHSIRP GLKAIIDKQI |
| Verpa_conica_No._21120 | 251 | 3.54e-55 | SPITLSIARE WYKNYEINMANDEREWRKLCFFPLTPFDTMLDWFTKARCRINSDTRAIRP AFKVIVDKAM |
| Verpa_conica_No._21117 | 231 | 3.54e-55 | SPITLSIARE WYKNYEINMANDEREWRKLCFFPLTPFDTMLDWFTKARCRINSDTRAIRP AFKVIVDKAM |

## Motif 2

| Name | Start | *p*-value | Sites |
|---|---|---|---|
| Morchella_importuna_[AVI61142] | 126 | 2.73e-60 | EEKIQKLQHN YTEDIWVAIADLHEFLIFACFVNQIHRDDTLDEKTLYSARNHLADSPHPE AGIALADLRA |
| Morchella_sextelata_[QQL94614] | 126 | 1.31e-59 | EKKIQKLQHN YTEDIWVAVADLHEFLIFACFVNQIHRDDTLDEATLYSAQNHLIDSPHPE AGIAFADLRA |
| Morchella_purpurascens_[QQL94647] | 126 | 6.27e-56 | EKKVQKLQHN YTEDIWVAIADLHEFLIFAYFVNQIHRGDTLDEETLYRAQNHLADSSHPE AGIAFADLRA |
| Verpa_conica_No._21120 | 156 | 1.22e-50 | LKILQTSIFK GEENIRVAIAEAHEFLLFATFINQLHRDDFFDDDTLQCATSHLEDSPHVE AAHALSGLRV |
| Verpa_conica_No._21117 | 136 | 1.22e-50 | LKILQTSIFK GEENIRVAIAEAHEFLLFATFINQLHRDDFFDDDTLQCATSHLEDSPHVE AAHALSGLRV |

## Motif 3

| Name | Start | *p*-value | Sites |
|---|---|---|---|
| Morchella_sextelata_[QQL94614] | 366 | 5.79e-62 | CTIDGNMKQA QHIIGRIFEAFTGTADFCMNVILRSDRLGGYEEKVFAQKSEKEFNIRYLS AVGEFLGQMR |
| Morchella_purpurascens_[QQL94647] | 366 | 1.76e-58 | CTIDGNMKHA QHTIGRIFEAFAGTADFCMNVILRSNRLGGCEEKLFAQKSEKEFNIRYLS AIGEFLGQRR |
| Morchella_importuna_[AVI61142] | 367 | 3.64e-57 | CTIDGNMKQI QHIIGRIFEAFTGTADFCMNAILHSDRLGGYEEKVFAQKSEKEFNIRYLS AVGEFLGQMR |
| Verpa_conica_No._21120 | 393 | 1.32e-45 | DSINPSIQEI QRLIKVIFESFATTAALSMKIILRDHGLDEVQEITSSQHSEREFNTRYLR YNVPVLNIVS |
| Verpa_conica_No._21117 | 373 | 1.32e-45 | DSINPSIQEI QRLIKVIFESFATTAALSMKIILRDHGLDEVQEITSSQHSEREFNTRYLR YNVPVLNIVS |

## Motif 4

| Name | Start | *p*-value | Sites |
|---|---|---|---|
| Morchella_purpurascens_[QQL94647] | 24 | 5.75e-57 | EATITPRKTT NRREAINQIRNVLCQVKNDLAYKPRAVPDAKIRDIAIFATRVLRRFRIKE LKRAIIDEKL |
| Morchella_importuna_[AVI61142] | 24 | 2.13e-56 | EATITPRKIT SRREAINKIRNVLCQVKNELAYKPRAVPDAKVRDVAIFSTRVLRRFRIKE LKRDSIDERL |
| Morchella_sextelata_[QQL94614] | 24 | 2.75e-55 | EATIIPRKTT SRREAISQIRNVLCQVKNDLAYKPRAVPDAKVRDVVIFSTRVLRRFRIKE LKRASIDERL |
| Verpa_conica_No._21120 | 51 | 3.01e-50 | LLTFIVANVS NRRKAIDRVVETLSQIRNELAYKPGAITDAKIRDVLLFALRVVRGFRVKY LRIKNLDETL |
| Verpa_conica_No._21117 | 31 | 3.01e-50 | LLTFIVANVS NRRKAIDRVVETLSQIRNELAYKPGAITDAKIRDVLLFALRVVRGFRVKY LRIKNLDETL |

## Motif 5

| Name | Start | *p*-value | Sites |
|---|---|---|---|
| Morchella_purpurascens_[QQL94647] | 291 | 1.93e-58 | IRDDSVVTKL NKGISMDEKRGETEKLDTLLADMIHEVDRLGSELEMKAFTPPPFPPSRAP QNHSPIKLQC |
| Morchella_sextelata_[QQL94614] | 291 | 1.99e-56 | IRDDSVVTKL NKGISMDEKRGETEKLDTLLADMIHEVDKLGSQLEMKAFTPPQFPPSCTP QNHSPIKLQY |
| Morchella_importuna_[AVI61142] | 291 | 1.78e-53 | IRDDSVVTKL NKGISMDEKRGETEKLDTLLADMIHEVDKLGSQLKMKAFTPPSFPPASCT PQNHSPIKLQ |
| Verpa_conica_No._21120 | 320 | 6.08e-46 | MKAKEVLAGF QKGQVKDDTRRDIEPINDLLKALILEVEGFGSLVKIRPFSPPPFPPSRAP ENKSPINLEY |
| Verpa_conica_No._21117 | 300 | 6.08e-46 | MKAKEVLAGF QKGQVKDDTRRDIEPINDLLKALILEVEGFGSLVKIRPFSPPPFPPSRAP ENKSPINLEY |

## Motif 6

| Name | Start | *p*-value | Sites |
|---|---|---|---|
| Morchella_importuna_[AVI61142] | 76 | 2.60e-56 | LRRFRIKELK RDSIDERLDFHPIADIYSLGHQVKSLLGAQSNLVRDLRYLEEKIQKLQHN YTEDIWVAIA |
| Morchella_sextelata_[QQL94614] | 76 | 5.78e-55 | LRRFRIKELK RASIDERLDFRPIADIYSLGHQVKSLLGAQSNLVRDLGYLEKKIQKLQHN YTEDIWVAVA |
| Morchella_purpurascens_[QQL94647] | 76 | 2.41e-51 | LRRFRIKELK RAIIDEKLDFHPIADIYSLGLQIKSLLGAQSILARDLGYLEKKVQKLQHN YTEDIWVAIA |
| Verpa_conica_No._21120 | 103 | 2.33e-42 | VRGFRVKYLR IKNLDETLDFYPLVNIHNLGRQIFSLFELQKKFTKSLRDVAIELKILQTS IFKGEENIRV |
| Verpa_conica_No._21117 | 83 | 2.33e-42 | VRGFRVKYLR IKNLDETLDFYPLVNIHNLGRQIFSLFELQKKFTKSLRDVAIELKILQTS IFKGEENIRV |

## Motif 7

| Name | Start | *p*-value | Sites |
|---|---|---|---|
| Morchella_sextelata_[QQL94614] | 455 | 7.68e-64 | PETRDLVLRD RHHSQILTCIFDAFGSLSEAEEEYLLGITHLHMSLEEIHEWFQKHSAKLQ RDNPEK |
| Morchella_importuna_[AVI61142] | 456 | 1.17e-63 | PETRNLILRD RHHSQILTCIFDAFGSLSEAEEEYLLGITHLHMSLEEIHEWFQKHSAKFQ NDKLEK |
| Morchella_purpurascens_[QQL94647] | 455 | 2.64e-62 | SETRGLFLRD RHNSQILTCIFDAFGSLSEAEEEYLLGVTHLHMSLEEIHEWFQKHSAKLQ SGNREK |

## Motif 8

| Name | Start | *p*-value | Sites |
|---|---|---|---|
| Morchella_sextelata_[QQL94614] | 342 | 2.60e-29 | PQFPPSCTPQ NHSPIKLQYWVSDTCTIDGNM KQAQHIIGRI |
| Morchella_importuna_[AVI61142] | 343 | 2.60e-29 | SFPPASCTPQ NHSPIKLQYWVSDTCTIDGNM KQIQHIIGRI |
| Morchella_purpurascens_[QQL94647] | 342 | 3.80e-28 | PPFPPSRAPQ NHSPIKLQCWVSDTCTIDGNM KHAQHTIGRI |
| Verpa_conica_No._21120 | 371 | 2.20e-19 | PPFPPSRAPE NKSPINLEYRISDSINPSIQE IQRLIKVIFE |
| Verpa_conica_No._21117 | 351 | 2.20e-19 | PPFPPSRAPE NKSPINLEYRISDSINPSIQE IQRLIKVIFE |

## Motif 9

| Name | Start | *p*-value | Sites |
|---|---|---|---|
| Morchella_sextelata_[QQL94614] | 176 | 4.60e-33 | NHLIDSPHPE AGIAFADLRAARQVEKELQKLSYGEGLPT VSKVIKLGPL |
| Morchella_importuna_[AVI61142] | 176 | 1.96e-32 | NHLADSPHPE AGIALADLRAARQVEKEFQKMNHGEGLPT VSKVIKLGPL |
| Morchella_purpurascens_[QQL94647] | 176 | 2.03e-31 | NHLADSSHPE AGIAFADLRAARQVEKELQKMSYDKGLPT VSGVIKFGPL |
| Verpa_conica_No._21120 | 206 | 1.27e-25 | SHLEDSPHVE AAHALSGLRVALSVSKELEKKDCRENPTV SSPTKLSPIT |
| Verpa_conica_No._21117 | 186 | 1.64e-25 | SHLEDSPHVE AAHALSGLRVALSVSKELEKKDCRETPTV SSPTKLSPIT |

Motif 10

| Name | Start | *p*-value | | Sites | |
|---|---|---|---|---|---|
| Morchella_sextelata_[QQL94614] | 420 | 2.93e-36 | NIRYLSAVGE | FLGQMRFHKVFEAINPDEFDSEELTPETR | DLVLRDRHHS |
| Morchella_importuna_[AVI61142] | 421 | 1.22e-35 | NIRYLSAVGE | FLGQMRFHQVFEAINPDEFGSKELTPETR | NLILRDRHHS |
| Morchella_purpurascens_[QQL94647] | 420 | 1.79e-30 | NIRYLSAIGE | FLGQRRFHKAFEAINPDELDSKGLTSETR | GLFLRDRHNS |
